# Depressive symptoms following traumatic brain injury are associated with resting-state functional connectivity

**DOI:** 10.1017/S0033291721004724

**Published:** 2023-04

**Authors:** Lizhu Luo, Christelle Langley, Laura Moreno-Lopez, Keith Kendrick, David K. Menon, Emmanuel A. Stamatakis, Barbara J. Sahakian

**Affiliations:** 1 Department of Psychiatry, University of Cambridge, Cambridge, CB2 0SZ, UK; 2 The Clinical Hospital of Chengdu Brain Science Institute, MOE Key Laboratory for Neuroinformation, University of Electronic Science and Technology of China, Chengdu 611731, PR China; 3 Division of Anaesthesia, University of Cambridge, Cambridge, CB2 0QQ, UK; 4 Department of Clinical Neurosciences, University of Cambridge, Cambridge, CB2 0QQ, UK

**Keywords:** Depression, functional connectivity, resting-state fMRI, traumatic brain injury

## Abstract

**Background:**

To determine whether depressive symptoms in traumatic brain injury (TBI) patients were associated with altered resting-state functional connectivity (rs-fc) or voxel-based morphology in brain regions involved in emotional regulation and associated with depression.

**Methods:**

In the present study, we examined 79 patients (57 males; age range = 17–70 years, M ± s.d. = 38 ± 16.13; BDI-II, M ± s.d. = 9.84 ± 8.67) with TBI. We used structural MRI and resting-state fMRI to examine whether there was a relationship between depression, as measured with the Beck Depression Inventory (BDI-II), and the voxel-based morphology or functional connectivity in regions previously identified as involved in emotional regulation in patients following TBI. Patients were at least 4 months post-TBI (M ± s.d. = 15.13 ± 11.67 months) and the severity of the injury included mild to severe cases [Glasgow Coma Scale (GCS), M ± s.d. = 6.87 ± 3.31].

**Results:**

Our results showed that BDI-II scores were unrelated to voxel-based morphology in the examined regions. We found a positive association between depression scores and rs-fc between limbic regions and cognitive control regions. Conversely, there was a negative association between depression scores and rs-fc between limbic and frontal regions involved in emotion regulation.

**Conclusion:**

These findings lead to a better understanding of the exact mechanisms that contribute to depression following TBI and better inform treatment decisions.

## Introduction

Traumatic brain injury (TBI) is a highly prevalent complex condition, affecting men and women of all ages and socioeconomic backgrounds worldwide (Nguyen et al., 2016). In the USA, TBI is the leading cause of death and disability in individuals under the age of 45, and is one of the best established environmental risk factors for increased incidence of late neurodegenerative diseases, including Alzheimer's disease (Savulich, Menon, Stamatakis, Pickard, & Sahakian, 2018). TBI is defined as an alteration in brain function, or other evidence of brain pathology, caused by an external force (Menon, Schwab, Wright, Maas, & Health, 2010). There is a spectrum of severity associated with TBI, which is clinically assessed by the Glasgow Coma Scale (GCS) (Teasdale & Jennett, 1974). Individuals with a GCS score of 3–8 are classified as having a severe TBI, 9–12 as moderate TBI, and 13–15 as mild TBI (Nguyen et al., 2016). Moreover, TBI presents a significant socioeconomic burden. Consequences of TBI range from physical disabilities to long-term cognitive, social and behavioural deficits, which may result in family disruption, restriction in community participation, loss of earnings and poor quality of life (Khan, Baguley, & Cameron, 2003). In 2010, the economic cost of TBI in the USA was estimated at $76.5billion. Even mild concussions, not requiring any form of hospitalisation, can be associated with significant costs such as disability or loss of work (Finkelstein, Corso, & Miller, 2006).

TBI can lead to symptoms such as impaired cognitive function, headaches, fatigue and depression. Depression following TBI is one of the most commonly reported and debilitating consequences. Moreover, it has been found to be associated with greater functional impairment and poorer recovery, poor psychosocial outcomes and decreased cognitive function (Rapoport, Mccullagh, Streiner, & Feinstein, 2003; Salmond, Chatfield, Menon, Pickard, & Sahakian, 2005). Kreutzer, Seel, and Gourley (2001) reported a prevalence of 41.9% for a depression diagnosis in a population of 722 TBI patients, providing a reliable estimate, which was greater than the rate among healthy participants. Despite this, depression following TBI is rarely assessed and often not treated.

Multiple studies have examined the neural substrates of depression, and have implicated the whole brain. Liu et al. (2017) showed that the precuneus was associated with recurrent depression, whereas the middle temporal gyrus may be associated with anhedonia experienced in depression (Yang et al., 2017). Many studies have specifically implicated brain regions associated with the processing of emotional stimuli, such as the anterior insula, thalamus, amygdala and anterior cingulate cortex (ACC) as well as brain regions involved in cognitive control such as the dorsolateral prefrontal cortex (dlPFC) and dorsal anterior cingulate cortex (dACC) (Diener et al., 2012). The dorsal and subgenual ACC (sgACC) have been shown to be functionally distinct, but both related to emotional processing. The dACC is involved in cognitive aspects of emotion regulation, while the sgACC is associated with emotional processing, specifically of negative stimuli, and has extensive connections with limbic regions and mood regulating cortical areas such as the orbital frontal cortex (Pandya, Altinay, Malone, & Anand, 2012). In addition, it is well established that patients with depression show reduced hippocampal and amygdala volume compared to controls (Campbell, Marriott, Nahmias, & MacQueen, 2004). Clark, Chamberlain, and Sahakian (2009) have suggested the enhanced bottom-up responses to emotional stimuli, from regions such as the amygdala and thalamus, and reduced top-down control, associated with the dlPFC, may play a role in depression. Subcortical regions also play a role in depression, Robinson, Cools, Carlisi, Sahakian, and Drevets (2012) found attenuated ventral striatal activity in response to unexpected reward in patients with depression. Drysdale et al. (2017) examined a large sample of over 300 depressed patients. Although the main aim of the study was to identify distinct neuropsychological biotypes, based on clinical symptoms, of depression from the resting-state functional connectivity (rs-fc), there were several regions that showed overlap regardless of clinical symptoms experienced. These included limbic, parietal and prefrontal regions associated with emotion regulation and cognitive control and have been selected as regions of interest (ROIs) in the current study.

Little is known about the neural substrates of depression in TBI specifically. It is not known why some individuals develop depression following TBI and others do not. Studies have shown abnormal rs-fc in networks associated with emotional regulation in TBI patients with depression (Moreno-López, Sahakian, Manktelow, Menon, & Stamatakis, 2016). Similarly, Han, Chapman, and Krawczyk (2015) showed that the rs-fc of the amygdala, in the emotional regulation network, was associated with depression in TBI patients. However, the sample size in Moreno-López et al. (2016) was small and Han et al. (2015) focused on amygdala connectivity. As such a large sample study examining the relationship between depression and TBI in a more extensive network of brain regions involved in emotional processing may better elucidate the neural substrates associated with depression following TBI.

Therefore, in the present study, we aimed to determine whether depressive symptoms in TBI patients were associated with altered rs-fc or voxel-based morphology in brain regions involved in emotional regulation and associated with depression. The limited studies discussed suggest that there is some overlap in the neural circuitry with non-TBI depression. Examining the mechanism of TBI depression in a large cohort may allow us to establish this firmly and may open avenues for treatment.

## Methods

### Participants

A total of 79 subjects (57 males; age range = 17–70 years, M ± s.d. = 38 ± 16.13) with TBI from the Addenbrooke's Neurosciences Critical Care Unit Follow-Up Clinic, the Addenbrooke's Traumatic Brain Injury Clinic and the Royal London Hospital Intensive Care Unit participated in this study. The exclusion criteria were (1) National Adult Reading Test (NART) <70, (2) Mini Mental State Exam (MMSE) <23, (3) left-handedness, (4) contraindications for MRI scanning, (5) pregnancy or nursing, (6) a physical disability that could prevent participants from completing the screening or scanning stages, (7) history of psychiatric or neurologic disorders including premorbid histories of depression or substance abuse and (8) the presence of focal lesions. All participants gave written informed consent before participating in the study as approved by the Cambridgeshire Research Ethics Committee in accordance with the Helsinki Declaration.

### Measurements

Patients suffered from brain injury, the severity of which was quantified by their GCS (Teasdale & Jennett, 1974); Injury Severity Score (ISS) (Baker, O'Neill, Haddon, & Long, 1974); and the Acute Physiology and Chronic Health Evaluation II (APACHE II) (Knaus, Draper, Wagner, & Zimmerman, 1985). Functional outcome was categorised using the Glasgow Outcome Scale (GOS) (Jennett & Bond, 1975). Depressive symptomatology was evaluated with, the widely used, Beck Depression Inventory (BDI-II) (Beck, Steer, Ball, & Ranieri, 1996). We did not assess whether participants had a clinical diagnosis of depression. Patients were at least 4 months post-TBI (M ± s.d. = 15.13 ± 11.67 months) and were not receiving any acute hospital interventions. Demographic and clinical characteristics of the patients are presented in Table 1.

**Table 1.** Demographical statistics and clinical measurements (*N* = 79)

Variables	Mean	s.d.
Age	38	16.13
Time since injury (TSI; /months)	15.13	11.67
National Adult Reading Test (NART)	109.73	10.26
Mini Mental State Exam (MMSE)	28.10	1.70
Beck Depression Inventory (BDI-II)	9.84	8.67
Glasgow Coma Scale (GCS)	6.87	3.31
Glasgow Outcome Scale (GOS)	4.25	0.84
Injury Severity Score (ISS)	26.47	14.35
Acute Physiology and Chronic Health Evaluation (APACHE II)	18.08	6.17

*Note.* APACHE II was not available for five subjects.

### MRI data acquisition

Participants were scanned on a Siemens Trio 3-Tesla MR system (Siemens AG, Munich, Germany) at the Wolfson Brain Imaging Centre of Addenbrooke's Hospital (Cambridge, UK). The imaging session started with a localiser followed by a high-resolution T1-weighted, magnetisation-prepared 180 degrees radio-frequency pulses and rapid gradient-echo (MPRAGE) structural scan (TR = 2300 ms, TE = 2.98 ms, TA = 9.14 min, flip angle = 9°, field of view read = 256 mm, voxel size = 1.0 × 1.0 × 1.0 mm, slices = 176). A total of 300 volumes of echo-planar images were acquired during a resting-state scan with the following parameters: TR = 2000 ms, TE = 30 ms, flip angle = 78°, FOV read = 192 mm, voxel size = 3.0 × 3.0 × 3.0 mm, number of slices = 32. Participants were instructed during the functional scan, to not think of anything in particular and to keep their eyes closed.

### MRI data preprocessing

Structural MRI data processing was performed in Matlab (R2014b, MathWorks, Inc., USA) using the Computational Anatomy Toolbox (CAT12; http://dbm.neuro.uni-jena.de/cat) based on the Statistical Parametric Mapping (SPM12; http://www.fil.ion.ucl.ac.uk/spm) software. The preprocessing used default settings illustrated in the toolbox manual (http://dbm.neuro.uni-jena.de/cat12/CAT12-Manual.pdf). SPM12 tissue probability maps were used for the initial spatial registration and segmentation. The participants' structural T1-weighted images were segmented into three tissue types: grey matter (GM), white matter and cerebrospinal fluid. A group-specific template based on all participants was created using the DARTEL algorithm (Ashburner, 2007). Participants' scans were then warped through a flow field which stored the deformation information. GM images were spatially normalised to Montreal Neurological Institute (MNI) space and quality of spatial normalisation was assessed by visual inspection. Finally, images were smoothed with an 8 mm FWHM Gaussian kernel. Total intracranial volume (TIV) was computed and used as a covariate on the second level.

The resting-state functional MRI data preprocessing was conducted using a standard pipeline in the CONN functional connectivity toolbox (v.18.b; www.nitrc.org/projects/conn) implemented in SPM12 and MATLAB. The pipeline comprised the following steps: removal of the first five scans to control for initial signal instability; functional realignment to correct for movement; slice-timing correction; identification of outlier scans for subsequent regression by means of the quality assurance/artefact rejection software art (http://www.nitrc.org/projects/artifact_detect); spatial normalisation to a standard echo-planar imaging template in the MNI space; and spatial smoothing with a Gaussian kernel of 6 mm FWHM. Images were corrected for physiological noise using CompCor (Behzadi, Restom, Liau, & Liu, 2007). Linear detrending was also applied, and the subject-specific denoised BOLD signal timeseries were band-pass filtered (0.01–0.08 Hz) to eliminate both low-frequency drift effects and high-frequency noise.

### Regions of interest (ROI) analyses

Following preprocessing, we adopted an ROI approach to carry out rs-fc analyses. In the light of findings from Drysdale et al. (2017), depression can be subdivided into four biotypes, of which the common neuroanatomical core of pathology encompasses areas spanning the insula, orbitofrontal cortex, ventromedial prefrontal cortex and multiple subcortical areas. Twenty-six ROIs were selected for this study based on the functional MRI study (Drysdale et al., 2017). The 26 ROIs were constructed using MarsBar (https://github.com/matthew-brett/marsbar), 6 mm radial spheres were centred at the coordinates presented in Table 2. ROIs were considered on both left and right hemispheres. The spherical ROIs were then imported for timeseries extraction into CONN. Subsequently, ROI to ROI functional connectivity was conducted in CONN in the first level, resulting in 325 different functional connections. The rs-fc between ROIs was measured using Pearson's correlation. To increase the normality and standardise the data for group comparison, a Fisher *z*-transform was conducted. These values were used to perform the correlation analyses with BDI-II scores.

**Table 2.** Names and Montreal Neurological Institute (MNI) coordinates of the ROIs on the right hemisphere

No.	Regions of interest	Abbreviations	MNI coordinates		
			*x*	*y*	*z*
1	Hippocampus	HIP	27	−15	−18
2	Parahippocampal gyrus	PHG	26	−40	−8
3	Amygdala	AMY	19	−2	−21
4	Thalamus	THA	2	−13	12
5	Ventral striatum	VS	31	−11	0
6	Middle temporal gyrus	MTG	56	−13	−10
7	Precuneus	PCU	15	−63	26
8	Anterior insula	aINS	37	1	−4
9	Dorsal anterior cingulate	dACC	10	22	27
10	Subgenual anterior cingulate	sgACC	4	15	−11
11	Orbital frontal cortex	OFC	24	32	−18
12	Ventromedial prefrontal cortex	vmPFC	8	42	−5
13	Dorsolateral prefrontal cortex	dlPFC	47	11	23

### Statistical analyses

Statistical analyses of the behavioural indices and demographic variables were conducted using SPSS Statistics 22 (Armonk, NY, USA: IBM Corp.).

To examine whether there were associations between depression scores (BDI-II) and brain structure, we carried out a multiple regression analysis using SPM12 at a voxel-wise significance threshold of *p* < 0.05 false discovery rate (FDR) corrected, which is a widely used technique in the literature. The model contained age, time since injury (TSI), GCS and TIV as covariates of no interest. The analysis was limited to the same 26 ROIs used for the functional analysis (Table 2), by applying an explicit mask, incorporating all the ROI spheres, to the analysis in SPM 12.

To elucidate the mechanism of depression post-TBI, associations between the depression scores and rs-fc were explored using non-parametric partial correlation analyses between BDI-II scores and standardised correlation coefficients between any two regions as dependent variables, covariates included GCS scores, age and TSI. These were computed with the Permutation Analysis of Linear Models toolbox (PALM, version alpha 109, https://fsl.fmrib.ox.ac.uk/fsl/fslwiki/PALM; two-tailed; number of permutations = 10 000; threshold of significance, *p* < 0.05, uncorrected) implemented in MATLAB. FDR correction was then performed at *q* = 0.05 on the uncorrected significant findings from the permutation test.

BrainNet Viewer (www.nitrc.org/projects/bnv/) was used to display a three-dimensional representation of our findings. Scatter plots were produced using ggscatter, ggarrange and ggexport (ggpubr package) in Rstudio (Version 1.2.1335; www.rstudio.com) which is an integrated development environment for the programming language R (www.R-project.org). To better present the partial correlation between BDI-II and each significant rs-fc, residuals of both variables were obtained separately through regression analysis by regressing out the effect of GCS, age and TSI. Residuals plus original mean values were then presented in the scatter plots.

## Results

### Clinical characteristics

Most of the cohort were classified as having sustained moderate to severe TBI based on the admission GCS: 47 out of 79 TBI patients had severe TBI (GCS < 8), 27 patients had moderate TBI (8 ⩽ GCS ⩽ 12), and only five patients had mild (GCS > 12) injuries. This was also confirmed by ISS scores, i.e. 63 of 79 patients had a major trauma (ISS > 15). Of the total study cohort, 57 had minor depression (BDI-II ⩽ 13), nine had mild depression (14 ⩽ BDI-II ⩽ 19), 11 had moderate depression (20 ⩽ BDI-II ⩽ 28), and two had severe depression (BDI-II ⩾ 29). Spearman correlation analysis showed no significant correlation between BDI and GCS scores (*r* = 0.146, *p* = 0.199). The majority of the cohort were not on medication (47 patients), 32 patients were on some form of medication, of these 11 were on antidepressants (one amitriptyline, seven citalopram, three unspecified antidepressant). We compared the functional connectivity between the group with antidepressant medication (*n* = 11) and the group without antidepressant medication (*n* = 68) and found no significant differences after correction for multiple comparisons. Therefore, the whole cohort was included in the analysis (see online Supplementary Material).

### Voxel-based morphometry

There were no significant linear relationships between level of depression and GM volumes of the 26 ROIs, having controlled for the effect of age, TSI, GCS and TIV, at *p* < 0.05 (FDR-corrected). It is worth noting that the results, even at the uncorrected level, did not reach significance.

### Functional connectivity

**Fig. 1.**
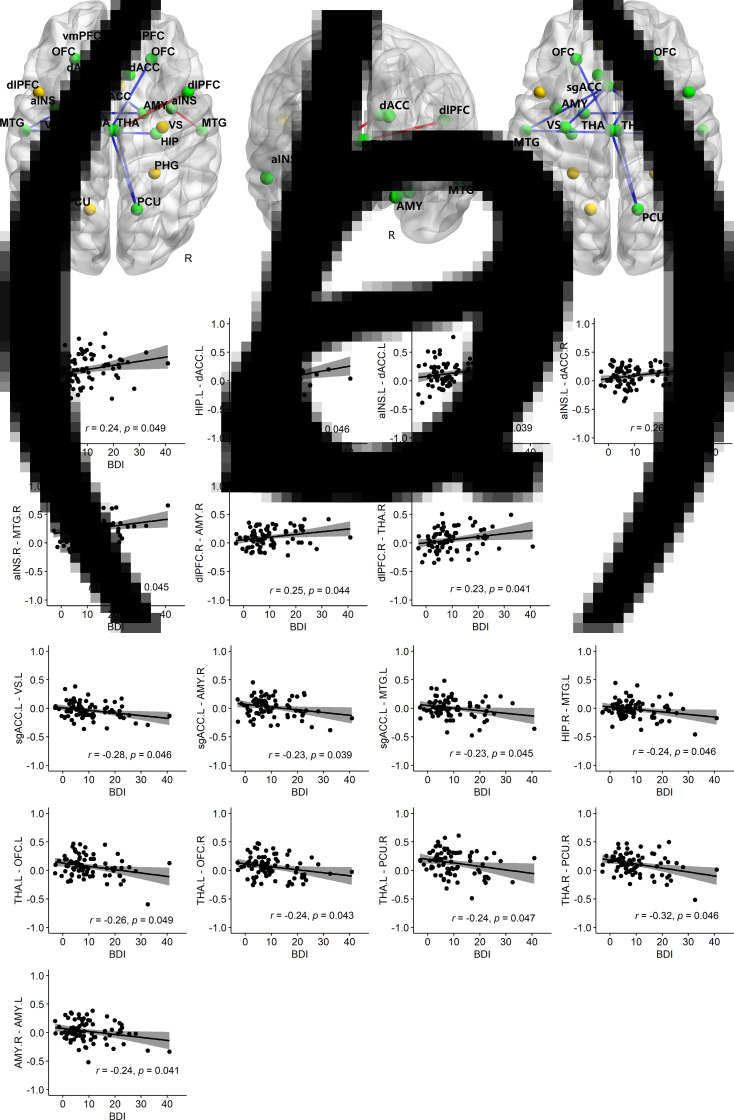
Non-parametric partial correlations between rs-fc and BDI-II, controlling for GCS, age and TSI. (a) Twenty-six ROIs are shown, represented by spheres. Yellow spheres denote regions with no significant correlations; green spheres show regions with significant correlations between the two regions' rs-fc and BDI-II. Red lines denote positive correlations with BDI-II; blue lines show negative correlations with BDI-II. The positive and negative correlations are shown together and separately in three brain maps. (b) Scatter plots of each significant correlation with original mean values plus residuals of BDI-II (*x*-axis)/rs-fc (*y*-axis) regressing out GCS, age and TSI. rs-fc, resting-state functional connectivity; BDI-II, Beck Depression Inventory; GCS, Glasgow Coma Scale; TSI, time since injury; *r*, partial correlation coefficient; *p*, FDR corrected. Confidence interval (CI), 95%.

We found significant linear relationships between level of depression and rs-fc in 19 of 325 paired ROIs (Figure 1), controlling the effect of GCS (we were interested in the broad picture across all injury levels), age and TSI (online Supplementary Table S1). Sixteen out of the 19 associations survived FDR correction. Functional connectivity between left HIP and both left AMY (*r* = 0.24, *p* = 0.049) and left dACC (*r* = 0.34, *p* = 0.046); left anterior INS and bilateral dACC (left, *r* = 0.23, *p* = 0.039; right, *r* = 0.26, *p* = 0.044); right anterior INS and right MTG (*r* = 0.27, *p* = 0.045); right dlPFC and both right AMY (*r* = 0.25, *p* = 0.044) and right THA (*r* = 0.23, *p*= 0.041) were found to be significantly positively associated with BDI-II scores.

BDI-II were significantly negatively associated with rs-fc between left sgACC and other three regions (left VS, *r* = −0.28, *p* = 0.046; left MTG, *r* = −0.23, *p* = 0.045; right AMY, *r* = −0.23, *p* = 0.039); left MTG and right HIP (*r* = −0.24, *p* = 0.046); left THA and bilateral OFC (left, *r* = −0.26, *p* = 0.049; right, *r* = −0.24, *p* = 0.043); right PCU and bilateral THA (left, *r* = −0.24, *p* = 0.047; right, *r* = −0.32, *p* = 0.046); and also between left and right AMY (*r* = −0.24, *p* = 0.041).

## Discussion

In the present study, we examined a large sample of patients with TBI, with a range of depressive symptoms, to determine whether depressive symptoms in TBI patients were associated with altered rs-fc patterns or voxel-based morphology in brain regions involved in emotional regulation and associated with depression. Our results showed that depressive symptoms were unrelated to voxel-based morphology, evidenced by no significant correlations between BDI-II scores and regional brain matter volumes. However, it is possible that the lack of correlations was due to the methodology used and further research is required to support this result. Severity of injury was also unrelated to depression scores. Indeed, previous studies (Seel, Macciocchi, & Kreutzer, 2010) have similarly shown no association between severity of injury and developing depression following TBI. We found altered rs-fc patterns, particularly in limbic regions, that were related to BDI-II scores. Our results showed a positive association between depression scores and rs-fc between limbic regions, and cognitive control regions. In contrast, there was a negative association between depression scores and rs-fc between limbic and frontal regions involved in emotion regulation (OFC and sgACC).

Our results also showed that increased rs-fc between the hippocampus and amygdala was associated positively with BDI-II scores. The hippocampus and amygdala are limbic regions, which are implicated in emotional processing. Previous studies have shown that reduced volume of the amygdala and hippocampus has been associated with depression (Campbell et al., 2004). In this large sample of TBI patients, there was no similar association between regional volume and depressive symptoms, but there was a positive correlation between BDI-II scores and the rs-fc between the hippocampus and the amygdala. As such it seems that in patients following TBI, depressive symptoms are related to dysfunctional communication between regions rather than their voxel-based morphology. The hippocampus is a key region for learning and memory and the amygdala is heavily involved in emotional processing. Neuroimaging studies in healthy adults (Smith, Stephan, Rugg, & Dolan, 2006) have suggested that amygdala-hippocampal connections modulate emotional memories. In depression, the increased amygdala-hippocampal connectivity was specifically associated with encoding of negative and not positive or neutral memories (Hamilton & Gotlib, 2008). It is therefore possible that the positive association between BDI-II scores and amygdala-hippocampal rs-fc may result from a bias towards negative emotional memories in patients following TBI.

Depression scores were positively correlated with rs-fc between the dlPFC and the thalamus and amygdala. The thalamus and amygdala are key regions in the cingulate-pallidostriatal-thalamic-amygdala mood-regulating circuit (Taber, Wen, Khan, & Hurley, 2004). Moreover, the thalamus has strong connections to the dlPFC, OFC and ACC and is therefore thought to play a vital role in emotional processing. The dlPFC, on the other hand, forms part of the central executive network and is involved in emotional processing through the top-down regulation of cognitive control. Functional imaging studies in patients with depression have often reported increased activation of the thalamus (Taber et al., 2004) and amygdala (Fales et al., 2008) and decreased activation in the dlPFC (Fales et al., 2008). Our results showed a positive relationship between BDI-II scores and rs-fc between the dlPFC and the amygdala and thalamus. This finding is interesting as studies often suggest a lack of cognitive control is associated with depression. Indeed, patients with TBI have shown compromised connectivity between the dlPFC and thalamus (Moreno-López et al., 2016), however this was not related to depression scores. The increased rs-fc of the dlPFC may be an attempt to upregulate cognitive control, but the strategy is ultimately ineffective. Moreover, studies have reported increased dlPFC activity in depression that is related to ruminations (Cooney, Joormann, Eugène, Dennis, & Gotlib, 2010). It may be possible that the positive correlation between depression and rs-fc between the dlPFC and thalamus and amygdala suggests that an inefficient strategy of cognitive control is associated with depression and increased rumination in patients following TBI. However, we did not measure rumination symptoms specifically.

Although the PFC is critical for cognitive control and executive function, several other structures contribute to cognitive control. The dACC, for example, is associated with cognitive aspects of emotion regulation (Pandya et al., 2012). Moreover, the dACC together with the insula forms part of the salience network, where the dACC is responsible for determining behaviourally relevant stimuli and guiding attention and the insula is responsible for the switching of attention (Menon & Uddin, 2010). In addition, the salience network has been associated with the integration of cognition and emotion for adaptive behaviour and has a high relevance for treatment response in depression (Diener et al., 2012). Our results showed a positive association between BDI-II scores and rs-fc between the dACC and anterior insula and hippocampus, further suggesting an inefficient strategy of cognitive control. The results from the current sample indicate an inefficient, rather than a compromised, cognitive control strategy that is associated with depression following TBI. This increased rs-fc may suggest an attempt to exert increased top-down control, although the strategy is ultimately ineffective.

We found a negative correlation between depression scores and rs-fc between the sgACC and a number of regions including the amygdala and ventral striatum. The sgACC has been strongly associated with emotional processing, specifically of negative stimuli (Pandya et al., 2012). Compared to controls, patients with depression demonstrated reduced resting-state connectivity of the sgACC (Drevets et al., 1997). The ventral striatum is a key region in the reward system, and due to its widespread cortical connections plays a role in decision-making (Haber, 2011). Consistent with our findings, a meta-analysis found decreased rs-fc between the sgACC and ventral striatum in patients with depression (Kaiser, Andrews-Hanna, Wager, & Pizzagalli, 2015). It is possible that due to the disconnection between these two regions, there is a lack of reward processing in the decision-making process. Together with the reduced rs-fc of the amygdala, the results suggest abnormal cortical-subcortical rs-fc that is associated with abnormal emotion processing and dysfunctional mood regulation.

Our results demonstrated a negative correlation with depression scores and rs-fc between the precuneus and thalamus and the thalamus and OFC. The OFC is key region in reward processing, specifically where increased activation is associated with increased reward expectation (Murray, O'Doherty, & Schoenbaum, 2007). The thalamus is a sensory integration hub in the brain and may act as a relay centre for functional brain networks (Hwang, Bertolero, Liu, & D'Esposito, 2017). The negative correlation between the rs-fc of these regions and depression may suggest that TBI patients with higher levels of depression do not adequately process the reward aspect of information. They may underestimate the expected reward which biases the decision-making process. Kim et al. (2017) argued that the disruption of this connection may be related to problems with decision-making and information integration in depressed patients. They demonstrated that reduced rs-fc between the thalamus and the OFC was related to suicidal ideation in patients with depression, possibly due to a reduced decision-making capacity. The precuneus is associated with highly integrative tasks such as self-awareness, visuospatial awareness and consciousness (Cavanna & Trimble, 2006). The negative correlation with BDI-II scores may suggest that the depression experienced by patients following TBI may be associated with dysfunctional self-monitoring and an inability to integrate information between subcortical and higher order cortical regions. Indeed, Malec, Testa, Rush, Brown, and Moessner (2007) demonstrated that self-awareness was associated with depression scores in patients following TBI. Specifically, focusing on perceived impairments was a key feature of increased reports of depression.

However, there are some limitations to the present study that need to be considered when interpreting the results. We did not have a control group with which to compare the TBI participants, as such we cannot directly state that these correlations are different from controls or unique following TBI. In addition, the interpretation of some of the rs-fc correlations is speculative due to the limited behavioural data and further research to explicitly test these interpretations is required.

Our results, from a large sample of TBI patients, suggest that altered resting-state rs-fc between neural substrates involved in cognitive control, emotion processing and the salience network may underlie the deficits in mood regulation following TBI. Our results showed three potential mechanisms that may contribute to the dysfunctional mood regulation: (i) increased amygdala-hippocampal rs-fc, which may suggest a bias towards negative emotional memories; (ii) an ineffective cognitive control strategy, which may be related to increased ruminations; and (iii) decreased integration between subcortical and higher order cortical regions, which may lead to decreased self-awareness and reduced decision-making capacity. These results demonstrated that a diverse network of regions are associated with depression in TBI. Future studies examining specific components of depression, for example, ruminations and affective bias, may be able to better elucidate specific neural substrates that underlie these components and also examine whether there are components that contribute more towards depression following TBI. In addition, the present study provides a better understanding of the neural mechanisms that may underlie depression following TBI. Our results support and extend those from previous studies (Han et al., 2015; Moreno-López et al., 2016). Further, our results showed overlap in terms of compromised connectivity in several regions often reported in depression without TBI, which include the amygdala, hippocampus, anterior insula, dACC, sgACC and dlPFC. However, further research directly comparing a depressed group with and without TBI would be required to statistically test the similarities and differences in the underlying neural mechanisms. In addition, studies including healthy controls would help to determine whether the correlations with rs-fc noted in the present study are altered. Previous studies comparing depressed and healthy individual have shown hyperactivity in regions such as the sgACC and amygdala in depressed patients (Cullen et al., 2016) and a similar hyperactivity may be present in depression following TBI. Similarly, Moreno-López et al. (2016) demonstrated that healthy controls showed correlations with depression scores in regions of cognitive control, not noted in TBI patients with depression, whereas the TBI patients showed correlations with depression scores in regions associated with emotion. This suggests that TBI patients may lack cognitive control over emotional cognition. Our results follow the same pattern, but we cannot statistically test this difference.

Depression following TBI is often underassessed and therefore not treated. Rehabilitation is often centred on physical therapy of movement and gait after injury often overlooks longer-term changes in mood and neuropsychiatric symptoms (Savulich et al., 2018). Wilson et al. (2020) found that mental health was associated with functional outcome in TBI patients, specifically in the mild cases. Together with our results, this highlights the importance of the assessment for depression following TBI. Selective serotonin reuptake inhibitors (SSRIs), which are the treatment of choice for depression, only show limited effectiveness in post-TBI depression (Kreitzer et al., 2019; Slowinski, Coetzer, & Byrne, 2019). A better understanding of the similarities and differences of neural mechanisms in people with depression with and without TBI may inform whether improvement of top-down cognitive control through cognitive behavioural therapy or transcranial magnetic stimulation or bottom-up improvements in mood and negative biases through pharmacological treatments including SSRIs may prove more advantageous (see Fig 1 from Roiser, Elliott, & Sahakian, 2012; see Fig 4 from Roiser & Sahakian, 2013).

## Supplementary material

For supplementary material accompanying this paper visit https://doi.org/10.1017/S0033291721004724. click here to view supplementary material
