# Supplementary material for: Depressive symptoms following traumatic brain injury are associated with resting-state functional connectivity
Source: Psychol Med. 2021 Dec 20;53(6):2698–705. doi: 10.1017/S0033291721004724 (PMC10123829; doi:10.1017/S0033291721004724)
Supplement: Supplementary file 1 [file S0033291721004724sup.zip › S0033291721004724sup002.docx]

**Supplementary Material**

As 11 participants were on antidepressant medication, we examined the differences between the group with antidepressant medication (n=11) and group without antidepressant medication (n=68) for BDI-II scores, MMSE, GCS and functional connectivity, despite the unequal group sizes. The BDI-II scores differed significantly (p<.01), which is expected given that those patients on antidepressant treatment are likely to have greater depression severity scores. There were no significant differences for MMSE or GCS scores. Importantly, there were no significant differences for the functional connectivity between the groups after correction for multiple comparisons. Therefore, we included the whole sample in the analysis.

**Supplementary Figure Legend**

**Supplementary Figure 1. Scatterplots of raw data between rs-fc and BDI-II.** Scatter plots of each significant correlation with original mean values of BDI-II (x-axis) / rs-fc (y-axis).

**Supplementary Table 1**. Significant correlations between rs-fc and BDI, controlling for GCS, age, and TSI

| ID | ROI1 | ROI2 | *r* | *p* | BH Adjusted *p* |
| --- | --- | --- | --- | --- | --- |
| 1 | L_HIP | L_AMY | 0.24 | 0.029 | 0.049 |
| 2 | L_HIP | L_dACC | 0.34 | 0.002 | 0.046 |
| 3 | L_aINS | L_dACC | 0.23 | 0.033 | 0.039 |
| 4 | L_aINS | R_dACC | 0.26 | 0.021 | 0.044 |
| 5 | R_aINS | R_MTG | 0.27 | 0.014 | 0.045 |
| 6 | R_dlPFC | R_THA | 0.23 | 0.037 | 0.041 |
| 7 | R_dlPFC | R_AMY | 0.25 | 0.023 | 0.044 |
| 8 | R_AMY | L_AMY | -0.24 | 0.032 | 0.041 |
| 9 | L_sgACC | L_MTG | -0.23 | 0.045 | 0.045 |
| 10 | L_sgACC | L_VS | -0.28 | 0.012 | 0.046 |
| 11 | L_sgACC | R_AMY | -0.23 | 0.037 | 0.039 |
| 12 | L_MTG | R_HIP | -0.24 | 0.031 | 0.046 |
| 13 | L_THA | L_OFC | -0.26 | 0.021 | 0.049 |
| 14 | L_THA | R_OFC | -0.24 | 0.032 | 0.043 |
| 15 | L_THA | R_PCU | -0.24 | 0.030 | 0.047 |
| 16 | R_THA | R_PCU | -0.32 | 0.005 | 0.046 |
| 17 | R_sgACC | L_MTG | -0.27 | 0.011 | 0.054 |
| 18 | R_AMY | L_OFC | -0.29 | 0.009 | 0.058 |
| 19 | R_aINS | R_vmPFC | -0.26 | 0.021 | 0.056 |

***Note:*** BH, Benjamini-Hochberg; ID 1-16, significant using an FDR of 0.05; ID 17-19, not significant.
