# Supplementary figures and images for: Depressive symptoms following traumatic brain injury are associated with resting-state functional connectivity
Source: Psychol Med. 2021 Dec 20;53(6):2698–705. doi: 10.1017/S0033291721004724 (PMC10123829; doi:10.1017/S0033291721004724)

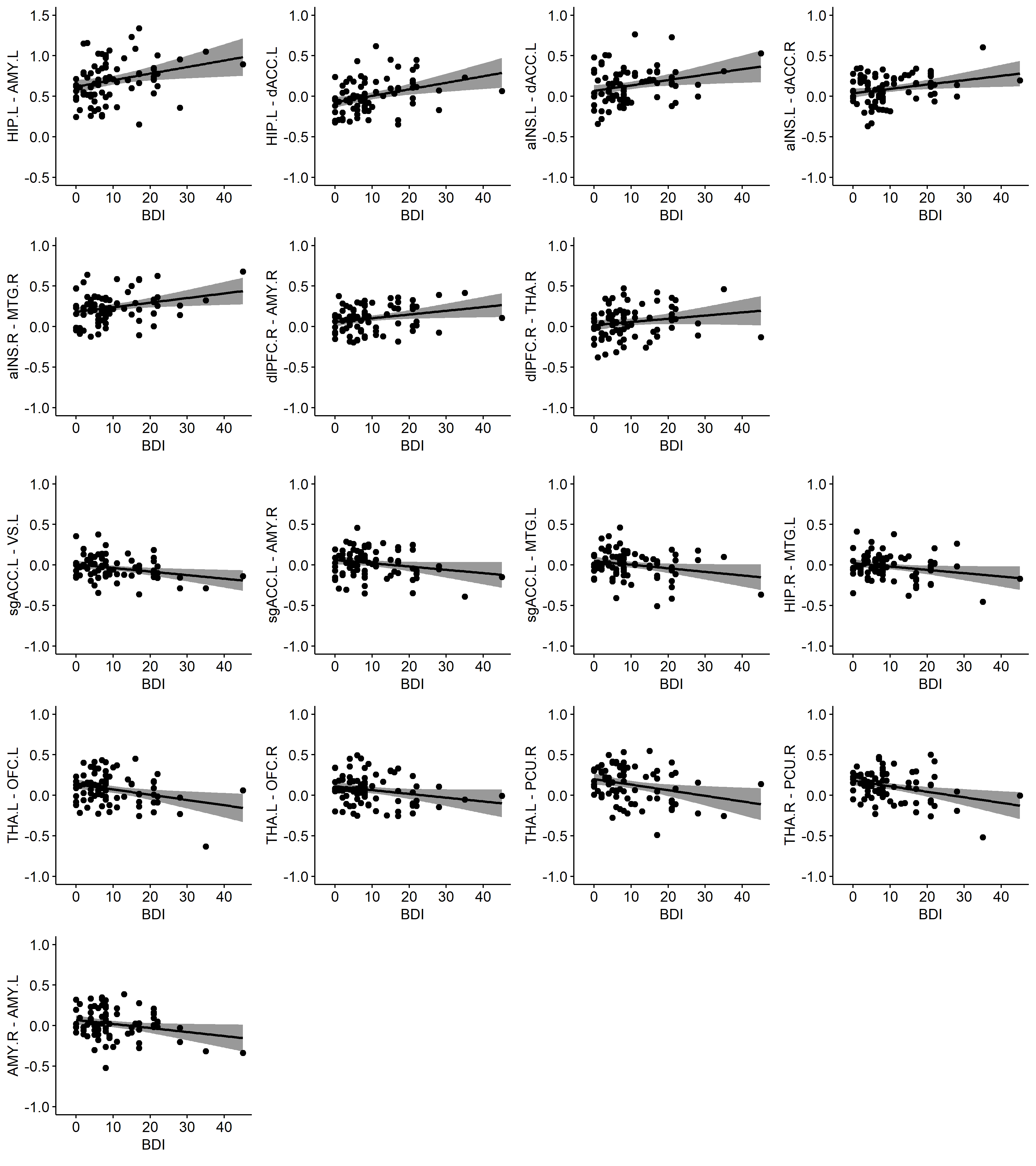

Supplement: Supplementary file 1 [file S0033291721004724sup.zip › S0033291721004724sup001.tif]
